# Barrett’s Esophagus: A Molecular Overview

**DOI:** 10.7759/cureus.3468

**Published:** 2018-10-19

**Authors:** Tatenda A Mudyanadzo

**Affiliations:** 1 Surgery, University of South Alabama, Mobile, USA

**Keywords:** barrett’s esophagus etiology, molecular changes barrett’s esophagus, genetic changes barrett’s esophagus, cell cycle, risk factors barrett’s esophagus

## Abstract

Barrett’s esophagus (BE) is an asymptomatic condition of the distal esophagus that can progress to aggressive adenocarcinoma of the esophagus. Although BE is not malignant, the amount of deoxyribonucleic acid (DNA) damage is comparable to some malignancies such as melanoma and breast carcinoma. The purpose of this literature review is to evaluate the anomalies that underlie the transformation of the normal stratified squamous epithelium of the esophagus into metaplastic columnar epithelium with a potential of progressing into esophageal adenocarcinoma based on an appraisal and scrutiny of the literature published since 2000. A systematic search of freely available journal articles pertinent to the pathoetiology (molecular and clinical risk factors) of BE was performed within PubMed and Google Scholar. All articles published in English reporting on the risks and molecular transformation of normal esophageal mucosa into metaplastic mucosa were considered; the research did not look further to the pathoetiology of esophageal adenocarcinoma. Each journal article was assessed based on the content, relevance, and applicability to this literature review. An assessment of 118 full-length articles produced 18 articles for the qualitative analysis. We noted risk factors, such as gastroesophageal reflux of acid and bile, cause aberrations at a molecular level to alter cell cycle control to culminate in morphological changes in esophageal mucosa, producing metaplastic cells with a potential of malignant transformation. There is a need for translational research to bridge the gap between genetics and molecular knowledge to achieve clinical preventive, diagnostic, and therapeutic approaches to addressing BE.

## Introduction and background

“The esophagus is part of the foregut, distal to the cricopharyngeal sphincter, which is lined by squamous epithelium [1]."  Norman Rupert Barrett, consultant surgeon at St. Thomas’ Hospital in London.

A 47-year-old obese woman presented with long-standing gastroesophageal reflux disease. She had a history of gastric banding and reported a history of esophageal ulcers. She presented with a request to have an esophageal examination. She had no dysphagia and had lost some weight since gastric banding. Upper endoscopy revealed white plaque in the posterior esophagus, and there were three tongues of abnormal-looking tissue extending 1 cm to 2 cm proximal to the Z line. Histopathologic examination revealed Barrett’s esophagus (BE). In this article, we discuss the risk factors and genetic abnormalities relating to BE.

Genetics and the cell cycle

The important role of cellular genetics in clinical medicine has been expanding rapidly. Testing fetal cells in the maternal circulation and testing cancer genetics to streamline chemotherapy are some of the vanguard applications of cellular genetics in medicine. An understanding of the cell cycle can lead to potential investigational and therapeutic areas of clinical medicine.

**Figure 1:**
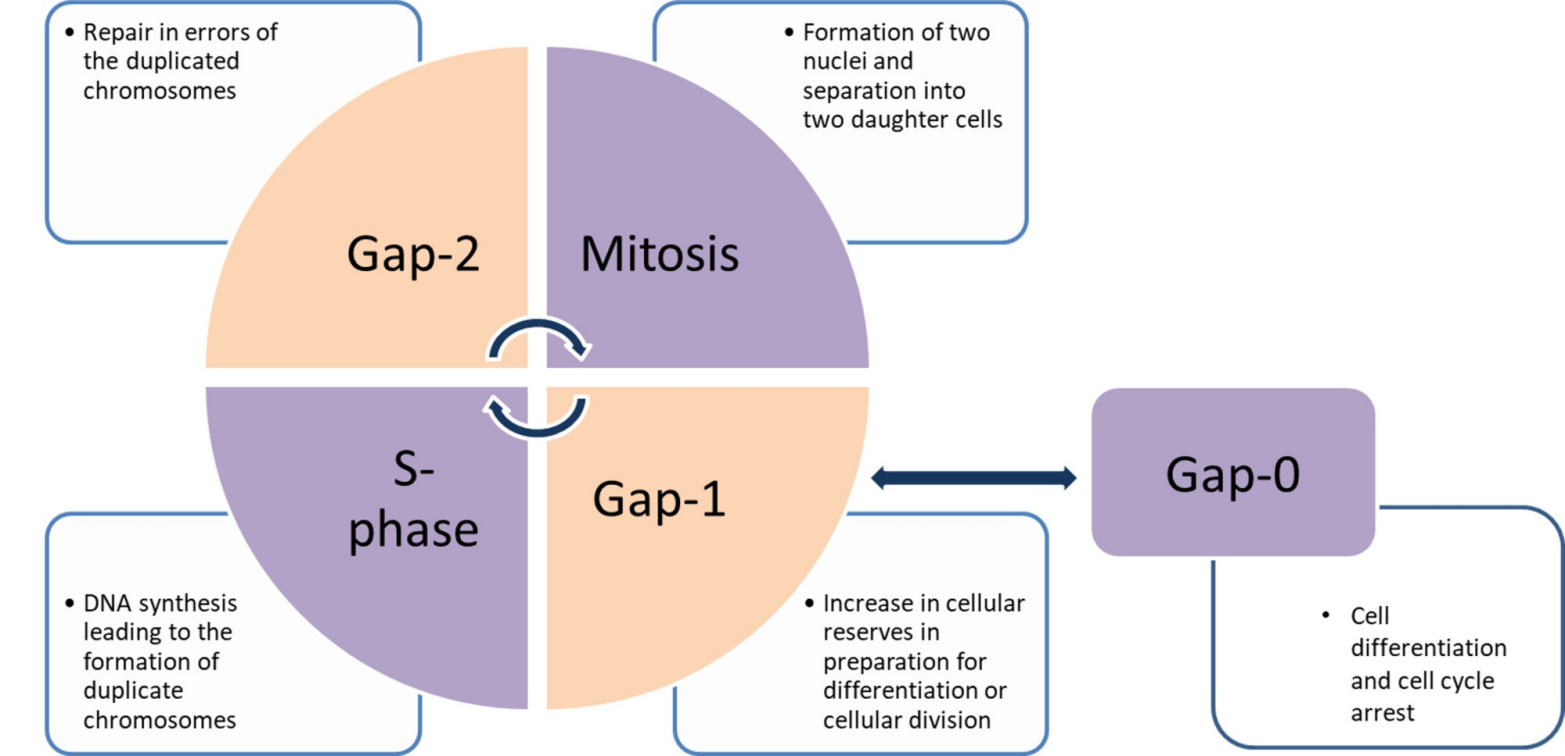
The cell cycle of events leading to the production of two daughter cells from a single parent cell

The cell cycle (Figure 1) is the sequence of events leading to the production of two daughter cells from a single parent cell; it involves deoxyribonucleic acid (DNA) replication and the division of the cell through processes of mitosis. Throughout this process, there are different points at which regulation occurs at checkpoints vital for ensuring the regulated growth of tissue. Loss of control at any of these checkpoints leads to excessive production of a cell lineage (i.e., cancer). Excessive control of the cell cycle checkpoints is related to the senescence of tissue cells as exemplified by aging.

The stages start at any point in the cycle; here, we will begin in stage Gap 0 (G0). G0 is a parent cell, a quiescent stable cell that is not involved in any alteration of its genetic content. Stimulation changes its state to enter G1 where the cell increases its replicative machinery and stores additional resources for the rest of the cycle. In this stage, the cell becomes larger and prepares for replication. To maintain the diploid nature of the cell, DNA must be replicated; this occurs in the S-phase of the cycle. There is a need to verify that all the prior cycle steps are done well before the parent cells produce two daughter cells; this verification step is the G2 phase. This is followed by the mitosis stage (M-stage) where cellular stores are expended to form two diploid daughter cells.

Errors in this cycle lead to mutations in the DNA of the daughter cell and, if unchecked, can lead to the transformation that underlies a variety of diseases, from heritable diseases to neoplasia. Alterations in the checkpoints of the cell cycle lead to cellular morphological changes in the pathogenesis of BE.

Barrett’s esophagus

BE is an asymptomatic acquired premalignant condition of the distal esophagus that predisposes to the development of esophageal and gastroesophageal adenocarcinoma (EA) [2-3]. BE is metaplasia from stratified squamous cells in the distal esophagus into a columnar-based epithelium, thereby bringing the squamocolumnar junction more proximal, with an irregular appearance, than the gastroesophageal junction; normally, these two junctions are found at the same level in healthy individuals [3]. The presence of BE confers approximately 30 to 40 times the increased risk of developing EA compared to patients without this lesion [3]. Endoscopy with biopsies and histopathologic examination are the modalities of choice for detecting and monitoring BE. Endoscopy, however, lacks sensitivity to be a tool utilizable for screening BE patients for the early detection and transformation into aggressive EA [3-4]. This has been attributed to the identification of BE, not rendering a decrease in the incidence and/or mortality related to EA. For those patients diagnosed with EA, a significant proportion of them did not have a prior diagnosis of BE, and approximately 40% of patients diagnosed with EA have no history of gastroesophageal reflux disease (GERD), and, thus, they had no prior endoscopic scrutiny [5].

The risk factors for developing BE are like those of EA. However, the opposite is not true. These risk factors include Caucasian race, male gender, age greater than 60 years, alcohol and smoking, obesity, history of GERD or heartburn, non-acid reflux disease, and genetic heterogeneity favoring familial BE [6].

## Review

Esophageal cancer has two main varieties: squamous cell cancer and adenocarcinoma. These two varieties of esophageal cancer are distinct entities with different cellular origins and pathoetiologies [7]. EA is associated with BE, and the pathological processes discussed here will relate to the adenocarcinoma variety and all references to cancer will relate to this variety.

Methods

The objective of this literature review is to assess the risk factors that may lead to the transformation of esophageal stratified squamous cells into columnar cells with a potential for malignant transformation. Individually, an assessment of PubMed and Google Scholar articles commenced on August 14, 2018, with the following terms used to locate resources relating to the pathology of BE: “Barrett’s esophagus etiology,” “molecular changes Barrett’s esophagus,” “genetic changes Barrett’s esophagus,” “cell cycle,” and “risk factors Barrett’s esophagus.” Included abstracts are in English; those that focused on esophageal cancer were not included. Further, each journal article was assessed based on the content, relevance, and applicability to this literature review. A total of 118 full-length articles up to the year 2018 produced 18 reports for qualitative analysis and discussion in this review.

Molecular origins of BE

The development of esophageal cancer has a high burden of mutation akin to cancers with an identifiable carcinogen such as melanoma and lung cancer [7]. Non-dysplastic BE has predictably lower mutations than esophageal cancer; strikingly, it has a higher burden of mutations than some malignancies such as breast cancer and multiple myeloma [7].

Mutation, loss, and/or inactivation of regulatory genes is responsible for the loss of control of the cell cycle, leading to a change in the esophageal lining into BE [8]. These areas of probable anomalies in BE include tumor suppressor genes, epigenetics, and oncogenes. Tumor suppressor genes include p53 and p16—these are genes that are responsible for encoding proteins whose function is to control cell division or to cause cell death. The loss of function of tumor suppressor genes can be due to a mutation leading to a non-functional protein or to a lack of the encoded protein (reduced amount to no production) culminating in the loss of control of the cell cycle and loss of apoptosis stimulus from the genes. P16, also known as CDKN2A, is the most important tumor suppressor gene in BE. Eighty percent of BE is associated with p16 anomalies, and these can arise from the hypermethylation of the promoter sequence of p16, loss of heterozygosity, and mutation of the p16 gene [9]. Inactivation of CDKN2A leads to genomic instability and the resultant uncontrolled cell multiplication. Whereas the alteration of p53 is found in dysplastic BE and esophageal cancer, its abnormal function points to grave prognosis. The presence of a mutated p53 tumor suppressor gene is not a feature of early stage non-dysplastic BE. Both genes are involved in the control of G1 to S-phase control by inducing apoptosis if DNA repair fails or by blocking the progression of cell division at this stage [7-8]. A fault with these genes leads to excessive cellular division, lack of cell death, and progression to malignancy. Abnormally functioning proteins encoded by both tumor suppressor genes will accumulate intracellularly to become detectable by immunohistochemical assays [10].

Changes in gene function that are heritable to meiotically and/or mitotically derived daughter cells without a change in DNA sequencing is termed epigenetics. Modification in epigenetic governors can disrupt normal gene expression and consequently lead to malignancy [11]. Homeobox (Hox) genes code for proteins that attach to the DNA and result in switching specific genes on and off within the DNA to determine the fate of specific cells. Caudal-type homeobox (Cdx)-1 and -2 are two Hox genes involved in small intestinal cell regulation. Their expression has been linked to a transformation of cell architecture from squamous to columnar cells [12]. This heralds the development of metaplasia in the esophagus [6], and this function is achieved via the hypermethylation of promoter sequences on tumor suppressor genes, leading to the inactivation of the latter. In vitro studies have not clearly delineated the role of these Hox genes in initiating BE. However, research has pointed to this effect by favoring intestinal type metaplasia [6].

Other cell cycle regulators include protooncogenes, such as Myc gene, and tumor suppressor genes, such as the adenomatous polyposis coli gene involved in cell cycle control. This review excludes a discussion of these regulators, as their importance is in EA they and are not routinely found in a BE analysis [10].

Pathology of BE

EA has risen in incidence approximately five-fold since the 1970s. It has a high mortality rate (more than 90% [9]), and the leading risk factor for its development is BE, which confers a 30-fold to 40-fold increase in risk compared to the general population [3,9]. BE is part of the continuum related to the metaplasia-dysplasia-adenocarcinoma sequence; this progression is associated with progressive genetic and epigenetic events that result in multiple aberrations in the cell cycle control and subsequently clonal selection to cause the emergence of esophageal cancer.

**Figure 2:**
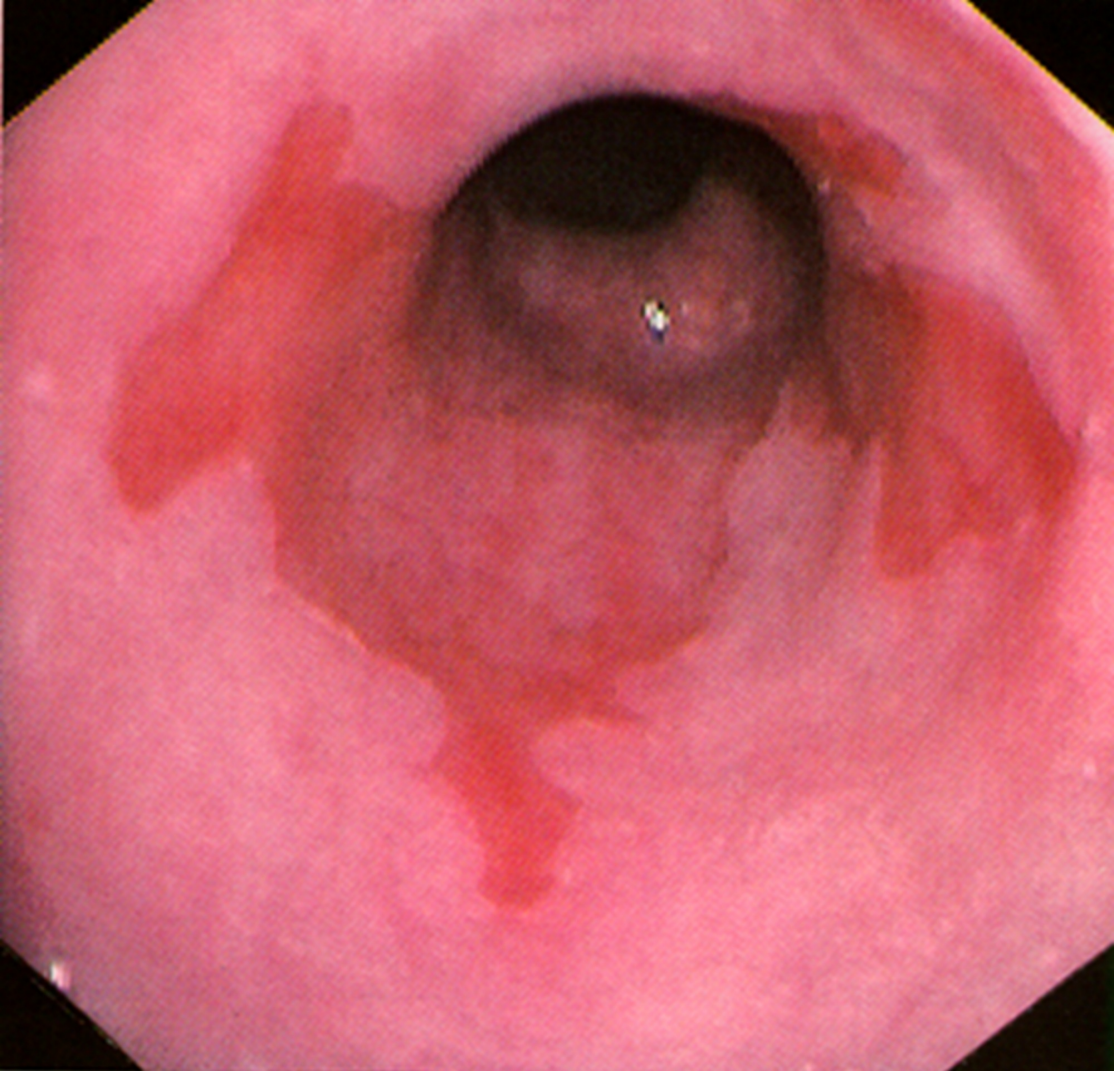
Endoscopic examination showing tongues of Barrett’s esophagus extending proximal to the gastroesophageal junction

BE is an acquired metaplasia where the normal squamous cells lining the esophagus are replaced by the salmon pink tongues of projecting lesions composed of columnar cells with an intestinal cell-like epithelium [9]. Diagnosis is by endoscopy and histopathology examination. The endoscopic examination revealed a separation of the squamocolumnar junction and the gastroesophageal junction along with color changes from glossy white to salmon pink (Figure 2). In clinical practice, immunohistochemistry and flow cytometry are rarely used; their use is related to research in delineating DNA damage and cellular changes related to the metaplasia-dysplasia-adenocarcinoma sequence.

The histopathology examination reveals columnar metaplasia with variations to the cell mosaic. The first type is a cardiac or junctional mucosa, which contains glands that are mucus producing and resemble the normal gastric cardiac type of cells. An atrophic variety that contains fundal type parietal and chief cells has a deeper glandular structure and secretes mucus like the first type. The last histological type contains a specialized intestinal type of cell that has crypts and contains goblet cells [8-9]. This variation in the type of cells found in BE types has caused some discord in the classification of BE; some entities support metaplasia and others choose a specific type of metaplasia to define BE. The British Society of Gastroenterology defines BE as any columnar metaplasia with intestinal type and/or cardiac-type mucosa whereas the American Gastroenterology Association traditionally labeled BE as columnar epithelium with an intestinal type of epithelium. The presence of goblet cells is the main differentiating factor between the cardiac type and the intestinal type of mucosa. Lately, there has been a shift of the definition of BE from a histological definition to a medical definition where BE is mucosal metaplasia with a possibility for a malignant transformation that replaces the normal squamous cells lining the distal esophagus [6,13].

Table 1 shows the risk factors and protective factors associated with BE [13].

**Table 1:** Risk factors and protective factors associated with BE Abbreviations: BE, Barrett’s esophagus; GERD, gastroesophageal reflux disease; NSAIDs, non-steroidal anti-inflammatory drugs; H. pylori, Helicobacter pylori

Factor	Risk factor for BE	Protective factor for BE
Age greater than 60 years	Yes	
Age less than 20 years		Yes
Caucasian race	Yes	
Male sex	Yes	
Hiatal hernia	Yes	
Family history of BE and esophageal cancer	Yes	
Obstructive sleep apnea	Yes	
Low birth weight for gestation	Yes	
Obesity	Yes	
Protracted GERD	Yes	
Consumption of red meat and processed meat	Yes	
Use of statins		Yes
Poor socioeconomic status	Yes	
Alcohol and tobacco use	Yes	
Use of NSAIDs		Yes
H. pylori infection		Yes
Diet rich in fruits and vegetables		Yes

BE develops through metaplasia. The columnar cells are thought to confer a protective effect from noxious stimuli to which the esophagus may be exposed. The metaplasia results from chronic esophagitis associated with GERD (i.e., gastric hydrochloric acid and reflux of bile and other noxious substances).

Etiology of BE

Gastroesophageal Reflux

The intermittent reflux of gastric acid and prolonged supine reflux conditions with a low esophageal pH are favorable conditions for the metaplastic changes in BE. Apart from normal gastric acid, new research indicates the importance of non-acid refluxate in the pathogenesis of BE. This includes the reflux of biliary salts, and deoxycholic acid is the most potent of these bile salts in inducing cell changes related to BE.

Bile salts and gastric acid are responsible for the generation of reactive oxygen species (ROS), which are involved in DNA damage as well as damage to progenitor cells linked to the regeneration of the esophageal epithelium [12]. Bile acids reduce the number of ROS scavengers and further enhance epithelial damage through cytokine-mediated action. In vitro experimental studies have shown the effects of bile and gastric acids being attenuated using antioxidants such as N-acetylcysteine and tempol. The antioxidants inhibit cytoplasmic and mitochondrial ROS generation at different levels, including the effect on nicotinamide adenine dinucleotide phosphate (NADPH) oxidases, which are involved in the production of ROS; this effect translates to reduced DNA damage, as noted by molecular testing [14].

Obesity

Obesity is related to multiple dysplastic conditions by favoring a pro-inflammatory state via enhanced quantities of interleukin-6 and interleukin-8. Centripetal obesity has been linked more to this factor due to the enhanced formation of these interleukins from adipocytes. The increase in intraabdominal pressure related to obesity enhances the reflux of gastric and non-gastric refluxate into the distal esophagus to initiate and perpetuate mucosal damage.

Diet

Diets rich in nitrates (vegetables, meat, and the secondary passage of ammonium nitrate from fertilizers used in agriculture) cause a high nitrate burden on the excretory pathways within the small intestines. Some of the nitrates get excreted in urine and others enter the circulation to be secreted by salivary glands where oral bacterial flora degrade this into nitrites. The gastric acid will act on these nitrites to form the highly reactive nitric oxide, which is genotoxic and carcinogenic [6]. Postprandially, some patients accumulate nitric oxide, enzymes (such as pepsin), and other noxious material near the gastroesophageal junction; these escape the dilution effects of ingested foodstuff, and they reflux into the distal esophagus to cause DNA damage through cytokine, ROS, and a direct effect on DNA [15-16].

The presence of ROS and nitric oxide offers a synergistic accentuation of mutagenicity via the production of peroxynitrite, which directly nitrates to form mutagenic DNA through DNA base aberrations [17].

Age, Race, and Sex

Male sex, increasing age, and Caucasian race are associated with BE. It is rare to find BE in pediatric patients, probably due to the lower incidence of other risk factors of BE and the enhanced efficacy of stem cells in this population, meaning they will repair any epithelial damage much faster than older patients. BE is most commonly found in patients older than 60 years [9].

Alcohol and Smoking

Nicotine has traditionally been considered a carcinogen, and research into its effects affirm this with regards to BE and esophageal cancer. Nicotine stimulates the production of ROS, is directly carcinogenic, and causes poor regeneration of blood vessels and vasoconstricts blood vessels. This latter effect can plausibly reduce the regenerative capacity of epithelial cells and will enhance the production of a low-oxygen tension environment that enhances stressful conditions on the damaged epithelium. The inflammatory background perpetuated by smoking impairs DNA repair mechanisms by inhibiting enzymes involved in DNA repair such as O-6-methylguanine-DNA methyltransferase [17]. The tissue hypoxia due to nicotine inhibits immunity directed at carcinogenesis and, at the same time, it advocates for the proliferation and angiogenesis that promotes carcinogenesis [17].

The correlation between smoking and esophageal cancer is strong with squamous cell esophageal cancer as compared to EA [15]. Wine consumption may have a protective effect on the pathogenesis of BE [6], however, inadequate literature was appraised to make any assertions regarding wine and BE.

Helicobacter pylori

Helicobacter (H.) pylori causes an atrophic effect on the gastric acid-producing cells, leading to a reduction in the production of gastric acid. This reduction translates to reduced risk to the metaplasia related to BE. H. pylori does not infect esophageal mucosa, and its carcinogenic effect has been confirmed with gastric carcinoma.

Familial Disease

Genetic predisposition has been linked to a familial type of BE [6].

Viral Infections

There are seven oncogenic viruses known to be involved in carcinogenesis. Among them, human papillomavirus, hepatitis, and Epstein-Barr virus are associated with gastrointestinal system malignancies. However, none of them are related to the etiology of BE [18].

## Conclusions

Metaplasia of the distal esophagus into columnar cells with a potential to progress into malignancy starts with molecular changes where there is a modification of genetic and epigenetic expression to cause dysfunction of tumor suppressor genes or proto-oncogenes such as the p16, p53, and Hox genes. These initiating events alter control of the cell cycle to confer the affected cells the ability to transform from one morphologic cell type to another with the possibility of progressing to malignancy.

Demographic and physical characteristics, such as age, sex, and weight, confer increased risk to the development of BE; they synergize with other risk factors, including reflux disease, to increase the risk of metaplasia and dysplasia. The challenge of predicting the transformation of BE into esophageal adenocarcinoma by serial endoscopy and the need of translating genomic and molecular abnormalities into a clinical screening, diagnostic, and therapeutic approach point to a need for more translational research into the area involving the metaplasia-dysplasia-adenocarcinoma sequence.
